# The Novice in General Practice

**Published:** 1919-08-30

**Authors:** 


### THE NOVICE IN GENERAL PRACTICE.

WITH the return of many of the younger medical generation to civilian life it may be expected that those among them who are now entering private practice for the first time will, after their initial experiences, remark, with even greater frequency than did the novices of pre-war days, that general practice differs disappointingly from their preconceived ideas; different from the service routine, where their medical ideals were guided by urgent military necessities; different in a thousand subtle details from the life in the great hospital to which they owe their training. Their new environment may suffer by comparison and distaste spoil their efforts, or, on the other hand, they may glide as into their natural element, but inevitably novices they are and must so remain until, with minimum loss of time and vacillation, they realise that although they now meet but rarely with pneumonias, typhoid fevers, or compound fractures, and although actually nine-tenths of their patients have relatively little the matter with them, yet many of this ninety per cent. represent as interesting and arresting material as can be brought to the notice of him who is a doctor but a student still.

Each may find his attention focussed more particularly on one or other type or temperament, but undoubtedly in the young practitioner such specialisation, moderate though it be, is best left unencouraged, and the first few years of his residence among strangers, strangers in a medical sense at least, devoted to detailed observation of all conditions of men which pass before him. Lately we have heard much of detecting very beginnings of disease, and no one is in a more favourable position to observe this essential part of our future health campaign than the general practitioner. But the day has passed when a young man followed for years the actual practice of his preceptor, learned disease pedigrees of whole families and the temperamental peculiarities of each neighbour before gradually relieving the older man of his local responsibilities. The path was clearly defined to this beginner, but nowadays, with fast-changing times and opinions, the novice is forced largely to accumulate his own store of knowledge, to limit his inexperience to a minimum period, and meanwhile to avoid the thousand pitfalls on the way.

It is impossible, even if we would, justifiably to urge a return to the old routine; but there is a method open to every beginner which should not only prove invaluable for his own guidance, education, and economy in time, but provide him with a wonderful knowledge of the community among whom his tent is pitched. Apart from developing keen power of observation and general receptivity, among the best means by which the novice may most rapidly acquire knowledge and understanding of this pseudo-medical side of general practice, we find the keeping, from the outset, of private individual and analytical records, not only from the purely medical view-point, but also with regard to temperamental and circumstantial details of every patient encountered. Memory is at best a poor retainer. Concise entries may be made by common symbols and contractions, and little greater demand made upon the doctor's time than with the usual medical diary. At least he will have notes, locally, of enormous future value to himself or a successor, and the experience gained in early stages of their compilation should aid materially in lessening the period of his novitiate.

